# Notch2 is involved in regulating apical tissue repair following severe intrusive luxation

**DOI:** 10.1590/1678-7765-2025-0490

**Published:** 2026-03-02

**Authors:** Yuanpei He, Xuyan Gong, Cancan Ding, Yang Qiu, Guangwen Li, Shiting Li

**Affiliations:** 1 The Affiliated Stomatological Hospital Southwest Medical University Luzhou China The Affiliated Stomatological Hospital, Southwest Medical University, Luzhou, China.; 2 Luzhou Key Laboratory of Oral & Maxillofacial Reconstruction and Regeneration Luzhou China Luzhou Key Laboratory of Oral & Maxillofacial Reconstruction and Regeneration, Luzhou, China.; 3 School of Stomatology & Affiliated Stomatological Hospital Tongji University Shanghai China School of Stomatology & Affiliated Stomatological Hospital, Tongji University, Shanghai.; 4 Shanghai Engineering Research Center for Dental Tissue Repair and Regeneration Shanghai China Shanghai Engineering Research Center for Dental Tissue Repair and Regeneration, Shanghai.

**Keywords:** Intrusive luxation, Notch signaling pathway, Apical repair, Angiogenesis, Fibrillization

## Abstract

This study aims to characterize apical repair dynamics in immature permanent teeth after severe intrusive luxation, and to explore Notch signaling pathway regulation in this process using a Sprague-Dawley rat model.

**Methodology:**

In this study, a modified tool was utilized to establish an intrusive luxation model in SD rats. Tissue repair was assessed via micro-computed tomography (Micro-CT) and hematoxylin-eosin (H&E) staining. Differential gene expression analysis of stem cells from the apical papilla (SCAPs) was performed using the publicly available GEO dataset. Immunohistochemical detection of Notch2 receptor expression in the apical region of injured teeth was subsequently conducted, guided by bioinformatics screening. Finally, the Notch signaling pathway was pharmacologically inhibited using DAPT (γ-secretase inhibitor), and its impact on post-injury prognosis was evaluated.

**Results:**

Histological analysis revealed prevalent pulp hypoplasia and apical tissue fibrosis after injury. Differential gene analysis suggested that the Notch signaling pathway, particularly Notch2, is involved in the regulation of angiogenesis-related pathways in SCAPs. Immunohistochemistry showed positive Notch2 expression in fibrotic apical regions. Notably, inhibition of Notch signaling significantly reduced aberrant fibrosis while enhancing vascular proliferation in the injury site.

**Conclusions:**

Severe intrusive luxation is primarily characterized by aberrant fibrous differentiation in the apical region. The Notch signaling pathway may negatively regulate angiogenesis in the damaged apical area, suggesting that targeted inhibition of this pathway could promote tissue repair following embedded dental injuries.

## Introduction

Intrusive luxation is a type of dental trauma where the tooth is displaced apically into the alveolar bone due to external force.^1^ Following intrusive luxation, spontaneous vascular pulp regeneration and natural re-eruption may occur.^2^ However, complications such as periodontal ligament ankylosis, pulp necrosis or infection, periapical periodontitis, root ankylosis, external replacement resorption, and inflammatory external resorption may cause adverse outcomes.^3^ This injury is prevalent in children and adolescents, as the dental pulp during this period exhibits stronger regenerative capacity.^4^ Preserving the pulp is critical for continued root development and apical foramen formation. Tooth loss can cause lifelong impacts on patients.^5^ Therefore, interventional treatment after intrusive luxation is particularly crucial during the young permanent dentition period.

Current research on intrusive luxation primarily relies on clinical case reports, with treatment approaches broadly categorized into three modalities: spontaneous re-eruption (SRE),^6^ orthodontic repositioning (ORP),^7^ or surgical repositioning (SRP).^8^ According to the 2020 International Association of Dental Traumatology (IADT) guidelines for dental trauma management, young permanent teeth affected by intrusive luxation can be monitored for SRE.^1^ If no re-eruption occurs within four weeks, orthodontic traction is recommended to activate pulp vascular regeneration. However, complications such as pulp necrosis, infection, or inflammatory external root resorption may necessitate root canal therapy. The underlying mechanisms remain poorly understood, particularly in experimental studies on intrusive luxation in young permanent teeth.^3,9,10^ To address this gap, Wang, et al. (2020) developed an experimental intrusive luxation model using a modified earring gun to apply force to the maxillary second molar of Sprague Dawley (SD) rats, facilitating investigations into the pathological and physiological processes of intrusive luxation and the factors influencing re-eruption.^3,11^ However, the embedded depth in their experimental setup remains debatable, and further validation is required to establish consistency with clinical intrusive luxation intrusion depths.

Regeneration of young permanent teeth after intrusive luxation is based on the pluripotency and developmental potential of stem cells in the developing dental pulp and apical tissues. In injured young permanent teeth, mechanical stress is directly transmitted to the underdeveloped root apex, potentially disrupting stem cell-mediated repair processes.^12^ Stem cells from apical papilla (SCAPs) and Hertwig’s epithelial root sheath (HERS) are critical sources during root development, playing pivotal roles in the formation and morphogenesis of dental roots.^13,14^ Periapical healing is dependent on the proliferative and differentiation potential of apical stem cells and the regulation of related biological factors. The Notch signaling pathway is one of the most important signaling pathways in tooth development and injury repair, and it is expressed at different stages in the development of dental tissues as well as during pulpal injury healing.^15,16^ Research indicates that the Notch pathway plays a regulatory role in SCAPs and influences HERS cells by promoting their proliferation and planar cell division, which is critical for root furcation formation.^17,18^ Among them, Notch2 receptor proteins play an important role. However, within the injured apical region, angiogenesis emerges as a pivotal process alongside odontogenic and osteogenic differentiation.^19,20^ Currently, no studies have specifically investigated the role of the Notch signaling pathway in the apical region following intrusive luxation.

Therefore, investigating the expression and regulatory role of the Notch signaling pathway during the re-eruption of young permanent teeth with intrusive luxation is crucial. In this study, a modified SD rat intrusive luxation model was utilized to analyze Notch2 receptor protein expression during the re-eruption process and assess changes in re-eruption under Notch signaling inhibition. The aim is to preliminarily clarify the relationship between the Notch signaling pathway and intrusive luxation prognosis, providing a theoretical basis for clinical intervention strategies.

## Methodology

### Experimental animals

A total of 32 male SD rats were used in this study (16 in the non-intervention group and 16 in the DAPT-treated group). SD rats (3 weeks old, mean weight 67.4±3 g) were sourced from Southwest Medical University’s animal facility. They were group-housed (4 per cage) in polycarbonate cages under standardized conditions: 12-hour light/dark cycles, 22 ± 1°C ambient temperature, and humidity control, with *ad libitum* food and water.

The sample size for this exploratory study was determined using the Resource Equation Approach, a validated method for animal studies when prior data for power analysis are unavailable.^21^ This method ensures that the degrees of freedom (DF) for the experimental error fall within the recommended 10 to 20 range. With the number of experimental groups (k) being 4, the appropriate sample size per group (n) was calculated using the formula n = DF/k + 1. This yielded a range of 4 to 6 animals per group. The group size selected was n=4, which satisfies the lower bound of this statistical recommendation while adhering to the ethical principle of Reduction under the 4R framework (Reduction, Replacement, Refinement, Responsibility), thereby minimizing animal use without compromising the scientific validity of this preliminary investigation.

### Material and instruments

Pentobarbital sodium (Sango, CHN); 4% paraformaldehyde (Sinopharm, CHN); paraffin (Leica, GER); Xylene (Sango, CHN); ethanol (Sango, CHN); Phosphate-Buffered Saline (PBS, Sango, CHN); H&E Stain Kit (Solarbio, CHN); 100X citrate antigen retrieval solution (Sango, CHN); Ethylenediaminetetraacetic acid (EDTA, pH 8.0, Sinopharm, CHN); Notch2 antibody (Abcam, USA); CD31 antibody (servicebio, CHN); Anti-rabbit IgG (cst, USA), SABC-POD kit (Boster Biological Technology, CHN); DAPT (Selleck, USA), Dimethyl Sulfoxide (DMSO, SIGMA, GER); corn oil (biosharp, CHN); Micro-CT (μCT50, SCANCO Medical AG, Fabrikweg2, CH-8306 Bruettisellen, Switzerland); Olympus light microscope (Tokyo, Japan).

### Experimental methods

#### Study Design

The experimental procedures involving animals were performed in compliance with ARRIVE guidelines and received ethical approval from the Institutional Animal Care and Use Committee (IACUC) at Southwest Medical University (Protocol No. 201912-7). Three-week-old male SD rats (n=16; mean body weight 90±5 g) were randomly assigned to four experimental groups (n=4/group). Experimental dental intrusion was induced using modified striking instrument under general anesthesia via intraperitoneal injection of 2% sodium pentobarbital (0.25 mL/100 g body weight). The rat’s head was fixed on a silicone pad to minimize errors during surgery. A standardized intrusion force was applied to the right maxillary second molar, with intrusion depth precisely controlled at 1.0mm using a digital caliper-linked micromanipulator. The left maxillary contralateral teeth served as non-injured controls. Four observation time intervals were selected: 24 hours, 3 days, 7 days, and 2 months post-operation. At each designated endpoint, rats were deeply anesthetized and sacrificed through transcardial perfusion using 4% paraformaldehyde. The maxillary alveolar bone specimens were harvested for fixation (48 hours). Subsequent decalcification was performed with 10% EDTA solution (pH 7.4) for 21 days before histological preparation. Sequential tissue sections (4 μm thickness) were prepared to evaluate spontaneous eruption.

## Morphological assessment by micro-CT

Male SD rats aged 21 days (n=4 per group) were anesthetized by intraperitoneal administration of 2% pentobarbital sodium solution at a 0.25 mL per 100 g of body mass dosage. Following induction of experimental embedded dental luxation in the right maxillary second molars, all rats underwent transcardial perfusion with 4% paraformaldehyde. Maxillary specimens were subsequently immersed in 4% paraformaldehyde for 48 hours. Soft tissues were carefully dissected, and maxillary alveolar bone tissues were subjected to micro-CT (μCT50, SCANCO Medical AG, Fabrikweg 2, CH-8306 Bruettisellen, Switzerland) for volumetric analysis.

## H&E staining

Sixteen SD rats (n=4 per time point) with experimentally induced embedded dental luxation were anesthetized and observed. After transcardial perfusion with 4% paraformaldehyde, maxillary specimens were dissected, fixed in 4% paraformaldehyde for 48 h, dehydrated through graded ethanol, embedded in paraffin, and sectioned into 4 μm-thick slices. Deparaffinized sections underwent H&E staining for histopathological evaluation. Stained slides were observed under microscope. Histological changes in dental pulp and periapical regions were analyzed.

## Bioinformatics and gene expression analysis

Publicly available transcriptomic data for stem cells from the apical papilla (SCAPs) and hematopoietic progenitor cells were obtained from the Gene Expression Omnibus (GEO) database (GSE149930, https://www.ncbi.nlm.nih.gov/geo/query/acc.cgi?acc=GSE149930). Differential expression analysis was performed using GEO2R, an online tool provided by NCBI that leverages the limma package in R for statistical analysis. Raw data were processed through background correction and quantile normalization, followed by log₂ transformation. Differentially expressed genes (DEGs) were identified based on the following criteria: |log₂ fold change| > 1 and adjusted p-value < 0.05 (Benjamini-Hochberg method for false discovery rate control).

To explore pathway-specific alterations, we focused on genes associated with the Notch signaling pathway (ID: hsa04330), as annotated in the Kyoto Encyclopedia of Genes and Genomes (KEGG) database. A heatmap was generated for these Notch-related DEGs using z-score normalized expression values to visualize expression patterns. Hierarchical clustering was performed using Euclidean distance and complete linkage. Additionally, to enhance biological interpretation, differentially expressed components of the Notch pathway were mapped onto the KEGG pathway diagram. Upregulated genes (log₂FC > 1) are highlighted in red, downregulated genes (log₂FC < −1) in blue (https://www.kegg.jp/kegg/pathway.html).

## Immunohistochemistry (IHC) staining

Maxillary tissue sections underwent sequential processing beginning with xylene dewaxing and ethanol gradient rehydration. After quenching endogenous peroxidase with 3% H₂O₂ (10 min) and PBS washes, antigen retrieval was performed in citrate buffer (95°C, 20 min). Blocking was achieved with 5% Bovine serum albumin (BSA) (SABC-POD kit, China) at 37°C for 30 min. Primary antibody incubation used rabbit anti-Notch2 (Abcam; 1:200 dilution) at 27°C for 1 h, followed by HRP-conjugated secondary atibody (cst, USA) for 30 min. Visualization employed DAB substrate with hematoxylin counterstaining, followed by standard dehydration and mounting procedures for light microscopic evaluation.

## DAPT inhibition intervention

A Notch signaling inhibitor, DAPT (GSI-IX; Selleck Chemicals, Houston, TX), dissolved in 3% DMSO + 97% corn oil, was intragingivally injected into the right maxillary second molars of SD rats at 2.5 mg/kg (100 μL/dose). Rats received injections once every 48 h for three consecutive days pre-injury. Experimental embedded dental luxation was induced, and specimens were collected at 24h, 3d, 1w, and 8w post-injury (n=4/group). Maxillary tissues underwent transcardial perfusion, decalcification, paraffin embedding, and sectioning. H&E staining, Notch2 and CD31 IHC were performed to evaluate periapical tissue regeneration and Notch pathway activation.

## Statistical analysis

Quantitative data from immunohistochemical staining are presented as the mean ± standard deviation (n=4). Given the small sample size, formal statistical tests for normality and homogeneity have limited power. Therefore, assumption checking was performed using a combined approach. Normality of residuals was primarily assessed by visual inspection of Q-Q plots and residual distributions, supplemented by the Shapiro-Wilk test where applicable. Homogeneity of variances was assessed by ensuring the ratio of the maximum-to-minimum group variance was less than 4:1 and by using the Brown-Forsythe test. If the data violated the assumption of normality, a logarithmic transformation was applied prior to analysis. For comparisons across four time points and two treatment groups, two-way ANOVA was used with Tukey’s post hoc test for multiple comparisons. All analyses were conducted using GraphPad Prism 10.2.1 (GraphPad Software, USA). Statistical significance was considered at p-value < 0.05.

## Results

### Establishment of a modified animal model for intrusive luxation

**Figure 1:**
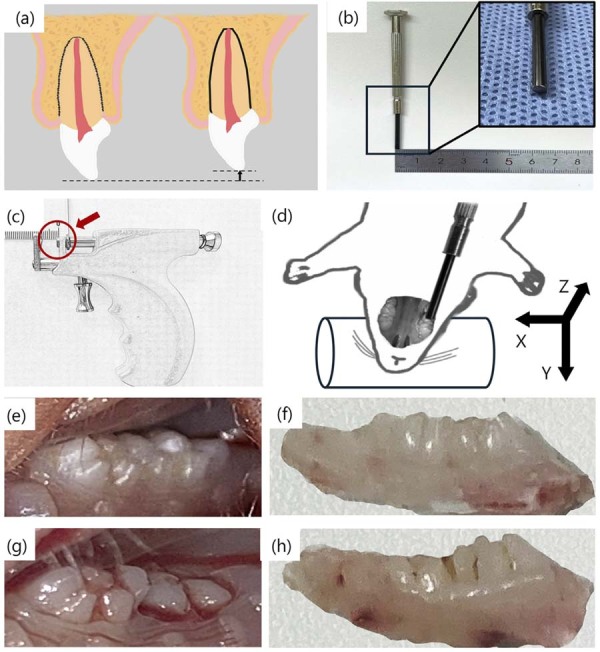
Establishment of intrusive luxation model with SD rats *in vivo*.

In SD rats (n=16), a modified pressure device was applied to the occlusal surface of the right maxillary second molar. The auricular forceps applicator was set to 1 mm intrusion depth. The contact tip of the guiding rod was precision-polished into a planar surface with a 2-mm diameter, corresponding to the functional anatomy of the maxillary second molar’s occlusal surface. This geometric congruence enables uniform stress distribution across the entire occlusal platform during force application. Following force application, the marginal ridge of the maxillary second molar descended from its original position flush with the marginal ridge of the maxillary first molar to the level of the gingival margin (Figure 1). Among the 16 rats, 15 achieved successful model establishment post-injury, while 1 rat died, confirming the reproducibility of this model. The deceased rat exhibited complete tooth avulsion accompanied by severe alveolar bone fracture and skull base perforation. The failure was likely associated with excessive manual force during device operation. Post-experimental observations revealed hyperplastic alveolar bone in partial specimens. Histological sections further identified pulp calcification, apical cysts, and external root resorption, potentially linked to an inflammatory microenvironment (Supplemental Figure 1).

(a) Schematic of intrusive luxation injury. Affected teeth exhibit reduced physiological crown height, periodontal ligament loss, increased periapical bone density, alveolar bone fracture, and apical tissue destruction. (b) A screwdriver tip was modified into a smoothed cylindrical structure (diameter: 2mm). (c) Custom instruments were calibrated to deliver a fixed intrusive force at 1mm depth. (d) Rat maxillary second molar intrusive luxation model. The right maxillary second molar was targeted, with the modified screwdriver tip aligned parallel to the tooth’s long axis for stable crown contact. (e, f) Preoperative view of the target molar. (g, h) Postoperative view following intrusive luxation.

**Figure 2:**
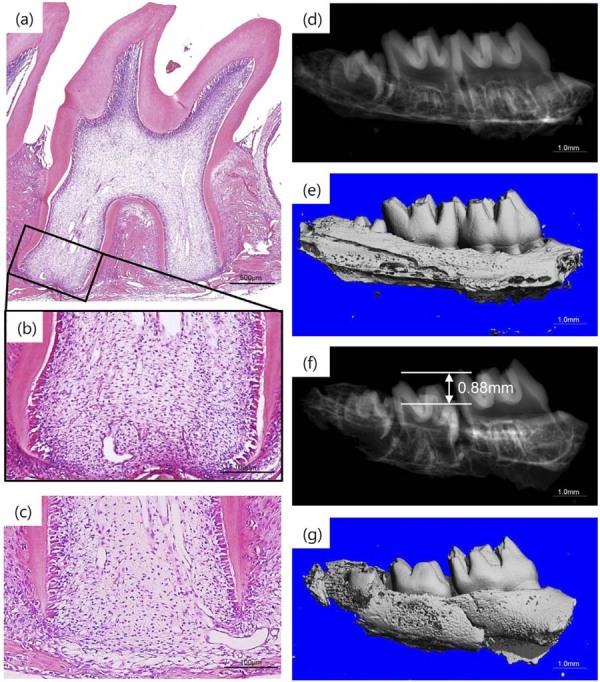
Histological and Micro-CT analysis of intrusive luxation.

Histological analysis revealed that the developing apical region exhibited parallel Hertwig’s epithelial root sheaths. Following intrusive luxation, the crowns remained intact without root fractures. Compression of the apical tissues was observed, leading to deformation of the developing Hertwig’s epithelial root sheath under external forces. Additionally, the alveolar bone in the apical region was disrupted by mechanical stress. Micro-CT imaging demonstrated vertical intrusion of the right maxillary second molar into the alveolar bone post-injury. The intrusion mobility depth measured 0.8–0.9 mm, attributed to the cushioning effect of the periodontal ligament. Radiographic findings confirmed preserved hard tissues without crown or root fractures, while alveolar bone fractures were identified, consistent with histological observations (Figure 2). The above results suggest that the area of influence of intrusive luxation is concentrated in the apical region, which has become the target of our attention.

(a, b) H&E staining revealed stress-induced bending of Hertwig's epithelial root sheath (HERS) tips in luxated teeth. Scale bars: 500μm(a), 100μm(b). (c) Uninjured control section (Scale bar: 100μm). (d, e) Micro-CT reconstruction of maxillary second molars in 3-week-old rats (Scale bar: 1.0mm). (f, g) Micro-CT quantification confirmed displacement of the second molar relative to the first molar.

### Notch2 expression in SCAPs

**Figure 3:**
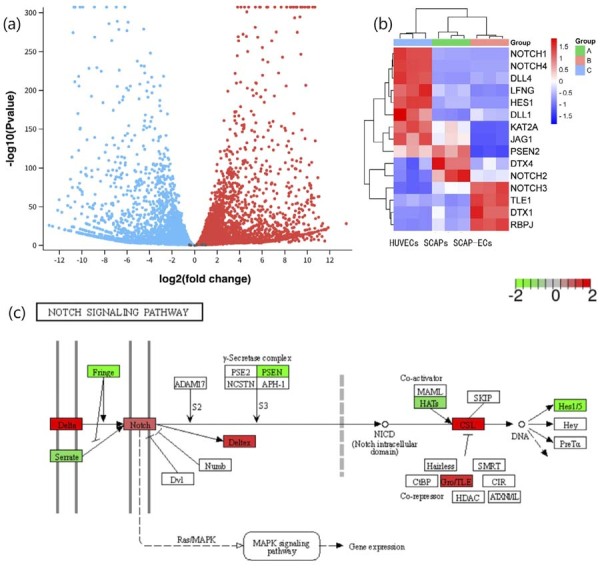
Transcriptomic analysis of stem cells from the apical papilla (SCAPs) in relation to the Notch signaling pathway (GSE149930).

In immature permanent teeth with open apices, SCAPs serve as pivotal cells for root elongation and pulp regeneration, and are closely associated with repair following apical injury. SCAPs exhibits distinct biological characteristics, with gene expression profiling revealing significantly higher Notch2 expression compared to coronal pulp.^22^ The process of injury repair often involves the regeneration of vascular-nerve bundles. Therefore, based on the GSE149930 dataset, this study conducted a comparative transcriptomic analysis between SCAPs and Human umbilical vein endothelial cells (HUVECs) with prominent pro-angiogenic properties, focusing on the expression profiles of NOTCH signaling receptors/ligands (Figure 3). Differential gene expression analysis revealed that the volcano plot delineated significant transcriptional disparities between SCAPs and HUVECs (|log2FC|≥1, FDR<0.05). Notably, heatmap visualization demonstrated a distinctive upregulation pattern of Notch2 in SCAPs, contrasting sharply with its expression levels in HUVECs and Endothelial cells of stem cells from apical papilla (SCAP-Ecs). KEGG pathway enrichment analysis further elucidated that Notch receptor families in SCAPs significantly suppressed angiogenesis-related pathways through a coordinated regulatory network during differentiation. These findings provide molecular mechanistic insights into the unique biological characteristics of SCAPs.

(a)Volcano plot showing differentially expressed genes (DEGs) between SCAPs and hematopoietic progenitor cells. Red dots represent upregulated genes (log_2_ fold change > 1), blue dots represent downregulated genes (log_2_ fold change < −1), with an adjusted p-value <0.05 (Benjamini-Hochberg correction). Statistical analysis was performed using the GEO2R platform (NCBI), which employs the limma package in R for linear modeling and empirical Bayes moderation. (b) Heatmap of Notch signaling pathway-related genes. Genes were selected based on KEGG pathway annotation (hsa04330). Expression values are shown as z-scores (scale: −1.5 to 1.5) derived from log_2_-transformed normalized data, with red indicating higher expression and blue lower expression across samples. Hierarchical clustering was performed using Euclidean distance and complete linkage. (c) KEGG pathway map (hsa04330) annotated to visualize expression changes in Notch pathway components. Genes significantly upregulated (|log_2_ fold change| > 1, adjusted p-value <0.05) are highlighted in red; downregulated genes are in blue. Gray labels indicate non-differentially expressed or undetected genes. The map was manually curated based on KEGG resources.

### Spatiotemporal correlation between apical repair and Notch2 receptor expression

**Figure 4:**
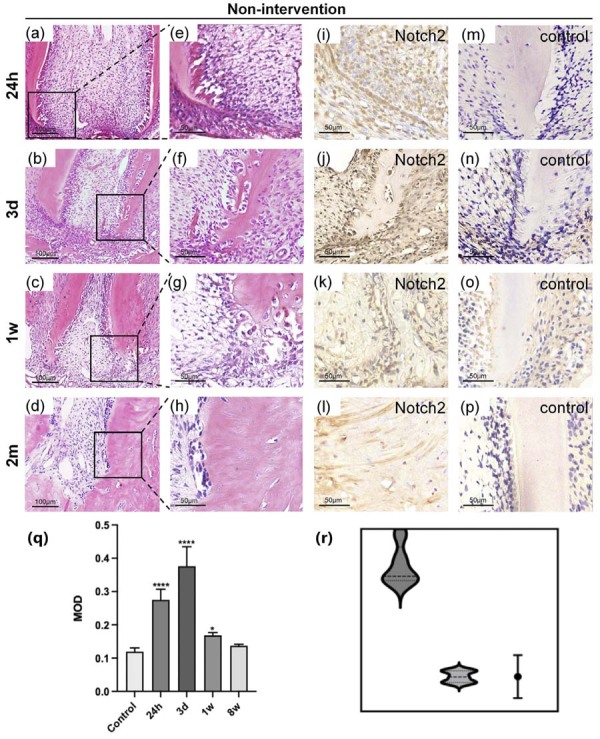
Histological findings of injury apical tissues.

Differential gene analysis identified Notch2 receptor protein as a key observation target following intrusive luxation. This evidence necessitates probing Notch2’s potential conserved role as a gatekeeper of apical regeneration. In SD rats, the maxillary second molar gradually re-erupted by week 4 post-trauma. However, apical tissue development was impaired after injury. Disorganized odontoblast layers and collagen replacement of normal periapical structures were observed in the apical region (Figure 4).

(a-h) HE staining of apical tissues after intrusion luxation. Magnification: ×20; scale bar: 100 μm(a-d). Magnification: ×40; scale bar: 50μm (e-h). (i-p) IHC staining magnification: ×40; scale bar: 50 μm. (q)The band intensities were analysed using ImageJ software. There was a statistically significant interaction between time and treatment group (F[3, 24] = 8.76, p<0.0001), as determined by two-way ANOVA.. The negative control for the IHC is the primary antibody omission control. The results represent the mean ± standard deviation of 4 independent experiments (n=4). *P<0.05, ****P<0.0001.(r)Comparing the violin plots of the two groups.

Within 24 hours post-injury, mechanical stress induces structural bending of HERS and disruption of the odontoblastic layer. Notch2-positive cells are specifically confined to the injured dental papilla niche. By third day, significant inflammatory infiltration occurs, with cellular aggregates mediating dentin resorption. These odontoclast-like cells exhibit intense Notch2 immunoreactivity. At first week postoperatively, pathologically resorbed dentin is replaced by collagen fibers, while Notch2 expression demonstrates spatially restricted downregulation in the inner HERS and pericervical regions. By second month, collagen matrix proportion increases significantly with concomitant cellular reduction and tissue hyalinization. Normal apical architecture is reconstituted by fibrotic disorganization, where Notch2 expression becomes markedly diminished. Collectively, Notch2 orchestrates apical microenvironmental remodeling following intrusive luxation through spatiotemporal modulation.

### Inhibition of Notch signaling enhances apical angiogenesis

To establish the causal role of Notch2 in apical tissue regeneration, we inhibited Notch signaling pharmacologically using DAPT. In SD rats receiving intragingival DAPT injections (2.5 mg/kg, initiated 3 days pre-injury and maintained at 48 hour intervals through post-injury week 1), accelerated tooth re-eruption occurred with a subset exhibiting taurodontism-like pulp chamber dilation. Critically, no osseous pathologies—such as alveolar osteomas or cysts—manifested in DAPT-treated specimens, distinct from the pathognomonic lesions observed in non-intervention cohorts (Supplementary Figure 1).

**Figure 5:**
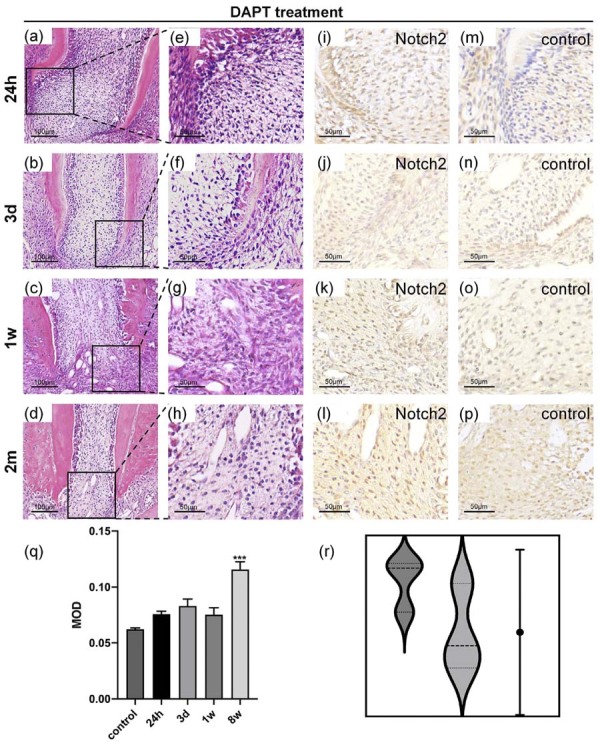
Inhibition of Notch signalling pathway expression with DAPT after intrusion luxation.

**Figure 6:**
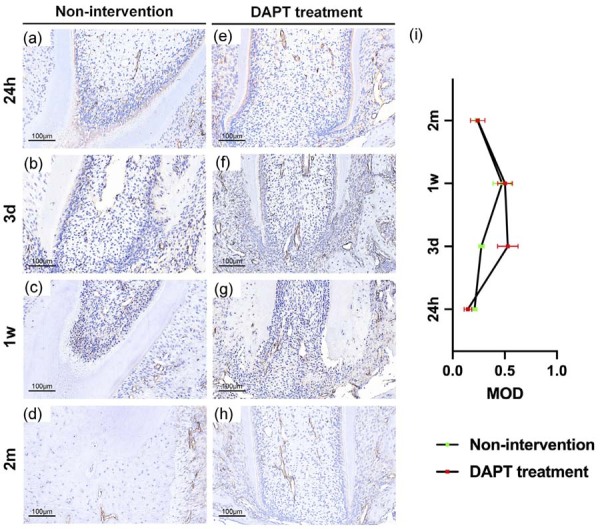
Expression of CD31 in the non-intervention group and the DAPT-treated group.

Attributable to DAPT intervention, Notch2 expression remained negative from injury initiation (Figure 5). Early histological alterations aligned with non-intervention controls. At third day post-injury, inflammatory infiltration diminished without pathological changes such as external root resorption in periapical tissues. CD31 expression was significantly enhanced, suggesting increased angiogenesis (Figure 6). By first week, extensive vascular bundle formation was observed in the apical region, with minimal development of pathological collagen fibrosis.

(a-h) HE staining of apical tissues after intrusion luxation. Magnification: ×20; scale bar: 100 μm(a-d). Magnification: ×40; scale bar: 50μm (e-h). (i-p) IHC staining magnification: ×40; scale bar: 50 μm. (q)The band intensities were analysed using ImageJ software. Two-way ANOVA revealed a significant interaction between time and treatment group(F[3, 24] = 9.42, p<0.0001). The negative control for the IHC is the primary antibody omission control. The results represent the mean ± standard deviation of 4 independent experiments (n=4). *P<0.05, ****P<0.0001.(r)Comparing the violin plots of the two groups.

(a-d) CD31 immunohistochemical staining showing vascularization in the injured apical tissue of the non-intervention group at 24hours, 3days, 1week, and 2 months post-injury. (e-h) Representative CD31 staining from rats treated with DAPT to inhibit Notch signaling, at corresponding time points. IHC staining magnification: ×20; scale bar: 100 μm.(i) Quantitative analysis was performed using ImageJ software. Data are presented as mean ± SD from four independent biological replicates (n=4 animals per group per time point). Two-way ANOVA revealed a significant interaction between time and treatment group (F[3, 24] = 12.45, p<0.0001), indicating that the temporal pattern of angiogenesis differed significantly between non-intervention and DAPT treatment groups. Post hoc multiple comparisons using Tukey’s test showed that the DAPT group exhibited significantly higher CD31^+^ area starting at day 3 (p<0.0005), with sustained elevation through 1 week.

Considering heightened regenerative activity in early-stage apical repair and potential systemic effects of DAPT on SD rats, interventions were ceased during the first week post-injury. By the second month, despite discontinued DAPT administration, early intervention yielded normalized apical morphology featuring mature vascular tissue and cellular collagen fiber formation along root surfaces. Significantly, periodontal ligament-like structures had regenerated surrounding the root. These results indicate Notch signaling likely exerts a negative regulatory effect on angiogenesis during apical repair—consistent with the differential gene analysis.

## Discussion

The 2020 IADT guidelines classified the severity of intrusive luxation and emphasized SRP when tooth intrusion exceeded 7 mm.^1^ Therefore, this study aimed to explore through an animal model whether surgery could be avoided or the vitality of the dental pulp preserved in cases of severe intrusive luxation. Wang, et al.^11^ (2020) successfully established an intrusive luxation rat model and observed tooth pathological changes consistent with human intrusive luxation. However, in the aforementioned experiment, the intrusion depth was set too deep, and the procedure may have caused death due to complications such as maxillary sinus perforation or skull base injury (10% mortality rate). To address this issue, we compared the dental anatomy between humans and rats and established a modified rat model that simulates severe intrusive luxation. Based on the anatomical comparison between human incisors and rat maxillary second molars, we determined that 0.8-0.9 mm was an appropriate intrusion depth.^23,24^ This avoided excessive force that could cause alveolar bone displacement or damage to the maxillary sinus floor (Table 1). Additionally, to ensure even pressure distribution from the guiding rod, we developed a 2 mm diameter force-transmitting rod with a flat tip (Figure 1).

**Table 1:** Comparison of intrusive luxation depths between human teeth and SD rat teeth

	Length of tooth	Mild	Moderate	Severe
Maxillary central incisor of human	23.5mm	<3mm	>3mm, <7mm	>7mm
Maxillary second molar of SD rat	2.24mm (2.10-2.38)	<0.3mm	>0.3mm,<0.7mm	>0.7mm

The dental pulp and apical papilla exhibit distinct Notch-related gene expression profiles. The mRNA levels of Notch2, MAML2, DTX4, and NEDD4 are significantly higher in the apical pulp complex compared to the coronal pulp tissue.^25^ These findings suggest that following severe intrusive luxation in rats, the stress-induced remodeling of the apical pulp complex disrupts the original signaling network in the immature root apex structure. SCAPs with differentiation potential are regulated to differentiate towards fibrosis or dystrophic calcification rather than dentin-pulp complex formation. Studies have shown that activating the Notch signaling pathway can induce osteogenic differentiation in SCAPs.^18,26^ Therefore, it is likely that mechanical stress following dental trauma activates the Notch signaling pathway, leading to the observed calcification in the apical region—this could serve as a target for further research.

One aspect that cannot be overlooked is that the compression and deformation of Hertwig’s epithelial root sheath (HERS) also represent significant histological changes. HERS cells play a crucial role in root apex development, exhibiting both epithelial and mesenchymal characteristics that promote the formation of periodontal ligament.^27^ Their potent osteogenic potential has also attracted researchers’ attention.^28^ Therefore, the extensive fibrous tissue observed in the root apex after injury may also result from dysregulation following HERS cell activation. Additionally, Notch2 is closely associated with HERS and plays a key role in molar furcation morphogenesis. Sun, et al.^17^ (2024) found that Notch2 expression was significantly downregulated in HERS cells of K14-Cre;Wnt10a mice. However, activating Notch signaling in K14-Cre;Wnt10a molars through Notch2 partially rescued the furcation developmental defects. Thus, it may be inferred that in our study, the downregulation of Notch2 following DAPT treatment led to furcation developmental defects, presenting as taurodontism-like changes, which is consistent with our findings (Supplementary Figure 1).

**Figure 7:**
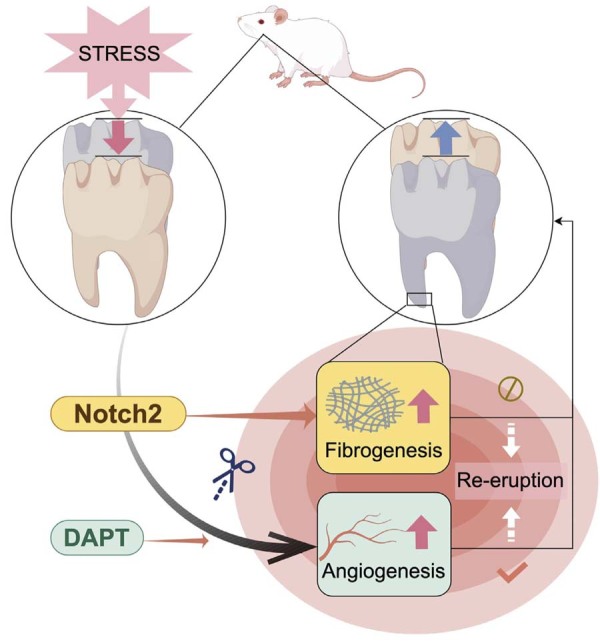
Schematic Illustration of Notch2 signaling-mediated regulation of tooth re-eruption in intrusion luxation rat model.

During dental injury repair, receptors and ligands of the Notch signaling pathway exhibit differential activation kinetics to critically modulate regeneration.^20, 29^ Notch2 is not expressed in intact adult teeth but is expressed in odontoblasts and preodontoblasts during dentin repair.^30^ *In vitro* studies have also found that human dental pulp stem cells express Notch2 during the early inflammatory phase.^31^ This means that during dental pulp repair, Notch2 is widely involved in the repair process across multiple cell types. Our study suggests that Notch2 may also be involved in the repair of the apical region (Figure 7). In addition to its potential role in suppressing angiogenesis, Notch2 may concurrently influence fibrogenesis, and its premature activation might disrupt the coordinated regeneration of vasculature and innervation in the apical papilla—a process critical for physiological tooth re-eruption. However, the functional link between localized vascularization and the re-emergence of immature teeth warrants further mechanistic exploration.

The mechanical stress led to intrusive luxation of the maxillary second molar in rats, as indicated by the tooth‘s movement toward the apex in the left circular inset. Following injury, endogenous Notch2 signaling modulated tissue regeneration within the inflammatory microenvironment of the apical region. Under physiological conditions, the rat apical region, regulated by Notch2, primarily exhibited fibrogenesis (represented by the gray mesh-like structure), a process unfavorable for tooth re-eruption. However, when the pharmacological inhibitor DAPT was applied, the blockade of Notch2 expression resulted in the upregulation of angiogenesis (depicted by the red branching structures). This enhancement in vascularization subsequently facilitated the tooth re-eruption, as illustrated by the tooth’s movement toward the crown in the right circular inset. (Graphic is created by figdraw.com. Copyright Code: RUIYUe10e3)

While findings suggest that Notch2 may suppress angiogenesis in the apical papilla, potentially impairing the coordinated tissue regeneration required for tooth re-eruption. We acknowledge that alternative mechanisms could also contribute to this process. For instance, the Notch signaling activation might exert indirect effects through modulation of local inflammatory responses. Emerging evidence indicates that Notch signaling can regulate macrophage polarization and cytokine production, which in turn influence both fibrogenic and angiogenic outcomes in regenerative contexts.^32,33^ Chronic or dysregulated inflammation may promote fibrotic scarring over functional vascularization, thereby compromising tissue remodeling in the periapical region.^34^ Additionally, mechanical load may interact with Notch2 activity. Studies have shown that mechanical strain can modulate Notch signaling components in stem cells, potentially altering their differentiation fate.^35, 36^ Although our current findings do not allow us to directly assess these interactions, the possibility of such indirect or synergistic mechanisms underscores the complexity of apical tissue regeneration. Future studies combining genetic perturbation with biomechanical or inflammatory challenge models will be essential to dissect cell-autonomous versus microenvironment-dependent effects.

## Conclusion

This study successfully established an optimized rat model using customized force applicators to simulate severe intrusive luxation, avoiding complications such as high-risk trauma and skull base injuries associated with anatomical constraints. Pathological analyses indicated that the Notch signaling pathway (particularly the Notch2 receptor) is involved in regulating tissue repair in the apical region, which is characterized by prominent fibrotic differentiation and an inverse correlation between Notch2 expression and vascularization. Inhibition of Notch signaling pathway reversed these pathological processes, notably enhancing neovascularization and reducing collagen fibrosis, thereby improving tooth re-eruption rates. These findings suggest that Notch signaling pathway provides a theoretical foundation for targeted interventions in severe intrusive luxation and imply that modulation of the Notch signaling pathway may represent a potential novel strategy to improve prognoses for such injuries.
